# IKN-CF: An Approach to Identify Key Nodes in Inter-Domain Routing Systems Based on Cascading Failures

**DOI:** 10.3390/e23111456

**Published:** 2021-11-02

**Authors:** Wendian Zhao, Yongjie Wang, Xinli Xiong, Jiazhen Zhao

**Affiliations:** 1 College of Electronic Engineering, National University of Defense Technology, Hefei 230037, China; zwd19@nudt.edu.cn (W.Z.); jiazhenzhao@nudt.edu.cn (J.Z.); 2 Anhui Province Key Laboratory of Cyberspace Security Situation Awareness and Evaluation, Hefei 230037, China

**Keywords:** inter-domain routing systems, complex network, key nodes, cascading failures, business relationships

## Abstract

Inter-domain routing systems is an important complex network in the Internet. Research on the vulnerability of inter-domain routing network nodes is of great support to the stable operation of the Internet. For the problem of node vulnerability, we proposed a method for identifying key nodes in inter-domain routing systems based on cascading failures (IKN-CF). Firstly, we analyzed the topology of inter-domain routing network and proposed an optimal valid path discovery algorithm considering business relationships. Then, the reason and propagation mechanism of cascading failure in the inter-domain routing network were analyzed, and we proposed two cascading indicators, which can approximate the impact of node failure on the network. After that, we established a key node identification model based on improved entropy weight TOPSIS (EWT), and the key node sequence in the network can be obtained through EWT calculation. We compared the existing three methods in two real inter-domain routing networks. The results indicate that the ranking results of IKN-CF are high accuracy, strong stability, and wide applicability. The accuracy of the top 100 nodes of the ranking result can reach 83.6%, which is at least 12.8% higher than the average accuracy of the existing three methods.

## 1. Introduction

With the continuous development of the Internet, the number of users on the network has grown extremely fast, and the scale of the inter-domain routing systems has continued to increase. An inter-domain routing system is a dynamic system composed of multiple autonomous systems (AS). It usually has four kinds of business relationships: customer-to-provider, provider-to-customer, peer-to-peer, and sibling-to-sibling [1]. AS generally refers to a collection of routers that belong to a management organization, and they can make their routing strategy. The routing information is exchanged between AS through the inter-domain routing protocol. Here it refers to the border gateway protocol (BGP). As the Internet’s only inter-domain routing protocol, BGP is the postal service of the Internet. AS plays the role of a branch of the post office. When users’ data requests were sent across the Internet, all of the data transmissions’ available paths were charged by BGP. It chooses the best route, making a hop between autonomous systems.

The BGP protocol greatly facilitates the transmission of information over the Internet. However, the inter-domain routing systems face many security threats because of the security defects of BGP [2,3,4,5,6]. The local failure caused by an intentional attack or self-failure of critical nodes in the network may continue to spread rapidly and wildly and eventually cause the whole network to be paralyzed on a large scale. This phenomenon is called a cascading failure. Some researchers have proposed a series of attacks that can cause the cascading failure of inter-domain routing systems, such as coordinated cross plane session termination (CXPST) [7] attacks, BGP stress attacks [8], and distributed network paralyzing (DNP) attacks [9]. This kind of attack mainly causes cascading failures of inter-domain routing systems by attacking key nodes and leading to colossal inter-domain routing system losses. Therefore, finding critical nodes in the inter-domain routing systems and implementing security policies in advance is of great significance to protect the inter-domain routing systems.

In previous studies, the importance of nodes was defined mainly by a single indicator, such as degree [10], betweenness [11], the clustering coefficient [12], and so on. In ordinary complex networks, this kind of method usually has better results. As for the inter-domain routing systems, due to its business relationships and dynamic characteristics, a single indicator cannot reflect the impact of node failure on the network. Then, some researchers added some indicators closely related to nodes based on a single indicator to assist in evaluating the importance of nodes, such as the substitutability of nodes [13], the connection ability of edges [14], the weak connection property of nodes [15], and so on. However, when multiple indicators are applied, the weight of each indicator is not considered, and the default value is the same. It is unreasonable. In addition, the importance value of nodes is obtained by simply adding multiple indicators or adding reciprocal sums, which cannot reflect the advantages of each indicator. In summary, the deficiencies of existing research are as follows:

(1) The indicator proposed by the researchers does not take into account cascading-failure processes in inter-domain routing systems.
(2) There is no reasonable method to calculate the weight of each indicator.
(3) Simple addition does not reflect the advantages of each indicator.

In order to better solve the existing problems in existing research, this study proposed a method for identifying key nodes in inter-domain routing systems based on cascading failures (IKN-CF). IKN-CF considers the cascading-failure characteristics of inter-domain routing systems and the business relationships, proposes two cascading indicators, and calculates the importance of nodes through improved the entropy weight TOPSIS (EWT) model. The results verify that the work in this study is meaningful. In general, the contributions of this study are as follows:

(1) We analyzed the topology of inter-domain routing networks and proposed an optimal valid path discovery algorithm considering business relationships.
(2) We analyzed the cause and propagation mechanism of cascading failures in the inter-domain routing network and proposed two cascading indicators, which can approximate the impact of node failure on the network.
(3) We established a key node identification model based on improved entropy weight TOPSIS, and the sequence of key nodes in the network can be obtained through model calculations.

The remainder of this article is arranged as follows: Section 2 introduces the preliminaries and related work. In Section 3, we analyze the topology of inter-domain routing systems and describe business relationships. Based on business relationships, an optimal routing path discovery algorithm is proposed. Section 4 introduces the implementation process of the IKN-CF method in detail. Experiments and analyses are described in Section 5. The last section summarizes the study and provides our conclusions.

## 2. Preliminaries and Related Work

This section explains the symbols used in the article and their corresponding meanings. Then, the related work is summarized.

### 2.1. Preliminaries

All parameters mentioned in this article are defined in Table 1.

### 2.2. Related Work

Some research has been done to look for key nodes in complex networks. Degree centrality [10] is the earliest proposed identification method for important nodes based on local features. Degree refers to the number of neighbors of a node. In this method, the greater the degree of the node, the more critical the node. Although the degree of complexity is low, the accuracy is not stable, and the phenomenon of the same order often occurs. Wang et al. [16] proposed a simple node importance evaluation method based on the degree of the node and the neighbor node. They believed that the degree of the node and the degree of neighbor nodes are greater and that the node is more important. Compared with degree centrality, this method can make full use of the local information of nodes to describe the differences between nodes. Later, researchers made improvements based on degree and proposed various methods to evaluate the importance of nodes [17,18,19,20,21]. Accuracy is increased to a certain extent, but complexity is also increased.

Freeman [11] proposed the concept of betweenness centrality based on global information when studying the influence of individuals in social networks. Betweenness refers to the number of shortest paths. The betweenness of a node usually refers to the number of shortest paths in the network that pass through the node. The number is greater and the importance of the node is higher in the network. Experiments show that the betweenness has high accuracy in identifying key nodes, but the complexity is also relatively high. Closeness centrality [22] is also a very typical method of identifying key nodes based on the global information of the network. The importance of a node is evaluated by calculating the shortest path length between the node and other nodes in the network. It is generally considered that the sum of the shortest path lengths is smaller and the closeness centrality is greater. It can be explained that the closer the node is to the center of the network, the more important the node is.

K-shell decomposition theory [23] is based on the key node method of node location attributes. The main idea of this method is to strip the nodes in the network from the outermost layer to the inner layer accordingly, removing one layer of nodes at a time. The last node to be removed is closer to the center of the network, which is considered to be more influential. The K-shell method is very suitable for large-scale networks due to its low complexity. However, there are limitations at the same time. The results of the classification of node importance are relatively rough in the network, and some nodes with large importance deviations are considered to have the same importance level. In addition, the K-shell method cannot distinguish the importance of nodes for special complex networks such as star networks and tree structure networks.

Recently, Ruan et al. [15] considered the characteristics of a weak connection of nodes and provided a method to evaluate the importance of nodes. Tang et al. [24] proposed the weighted K-order propagation number (WKPN) algorithm based on the network topology. Zhao et al. [13] proposed an edge-based and local partition method to identify the key nodes. Since the inter-domain routing systems have business relationships and are dynamic systems, the above method does not apply.

For inter-domain routing systems, there are few studies on identifying key nodes. Guo et al. [25] used degree as an index to evaluate the importance of nodes when studying the cascading failures process of the inter-domain routing systems. It is believed that a node with a higher degree has more adjacent nodes, which leads to a larger traffic. Therefore, it is believed that a node with a higher degree of removal will have a greater impact. Schuchard et al. [7] used betweenness as an index to evaluate the importance of edges when studying CXPST attacks. It is believed that the betweenness is larger and the edge is more important. This evaluation method has a certain accuracy, but routing selection not only relies on the shortest path between two nodes but also needs to integrate various influencing factors in the inter-domain routing systems. Typical factors include the business relationships between autonomous domains, local priority, the source address type, and the address learning source. Therefore, the shortest path is not necessarily the optimal path ultimately selected.

Liu et al. [26] believed that the betweenness could not represent the characteristics of the inter-domain routing systems. Under the premise of considering the business relationships, they proposed a technique of evaluating AS importance based on the preferred route (AIPR). The preferred route represents the number of the best paths through nodes. The preferred route solves the problem that the betweenness does not, meaning the actual traffic. However, it does not consider the cascading-failures process and cannot fully reflect the role of nodes in failure propagation. Zhu et al. [27] thought that the inter-domain routing systems are a dynamic process and proposed a method for identifying key nodes of the inter-domain routing systems based on propagation dynamics. The attributes presented by this method consider the process of cascading failures, but there is no reasonable method to define the weights between the features. In addition, the important value of the node is calculated according to the influence of the failure, which is unrealistic in the existing inter-domain routing systems.

## 3. Topology Analysis of Inter-Domain Routing Systems

The topology of inter-domain routing systems conforms to the general rules of complex network, such as scale-free characteristics and small-world characteristics. However, the inter-domain routing systems has business relationships that are not available in general complex networks, resulting in the path of the inter-domain routing network being divided into two types, namely, valid paths and invalid paths.

### 3.1. Business Relationships

A business relationship is a paid relationship in which customers can exchange traffic with the service provider only if they pay the service provider. There are complex business relationships in the inter-domain routing systems. For the inter-domain routing network, even if two nodes have a connection relationships but no business relationships, then the information cannot be transmitted.

Service providers play an important role in the Internet, providing new users with access to the Internet services. Business relationships can be summarized into three types: provider-to-customer (P2C) or C2P, peer-to-peer (P2P), and sibling-to-sibling (S2S) [28]. S2S only occurs in a few cases, and in order to make the study more general, S2S was not considered in this study.

**Figure 1:**
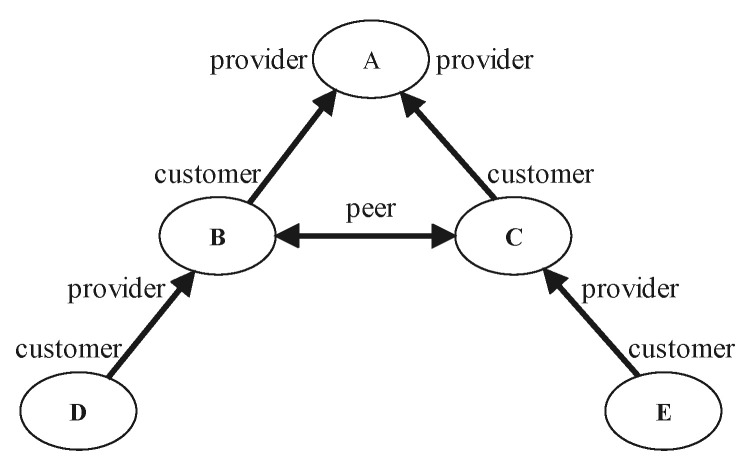
Business relationships.

The business relationships is shown in the Figure 1, with the arrow of the line in the direction of payment. For example, B→A means that customer B pays provider A, and B is a customer of A, that is, a C2P. A is the provider of B, that is, the P2C. At this time, customer B can exchange information with A to obtain resources. Similarly, customer D has to pay B to access resources. For B↔C, it is a peer relationship, that is, a P2P. The relationship between the two ends of the connection is equal, and information can usually be exchanged for free. Therefore, to exchange information between nodes of an inter-domain routing systems, links and business relationships are required. Otherwise, the path may be invalid. The following examples illustrate valid and invalid paths.

Article [28] gives the definitions of a valid path and an invalid path in inter-domain routing systems. In the path from the source AS node to the target AS node, each relay AS node has a customer connected to it. Such a path is called a valid path. Contrary to the valid path, at least one relay AS node does not pay customers to it. Such a path is called an invalid path. Therefore, the key to determining whether it is a valid path is to find all relay AS nodes and determine whether there are customers connected to them.

**Figure 2:**
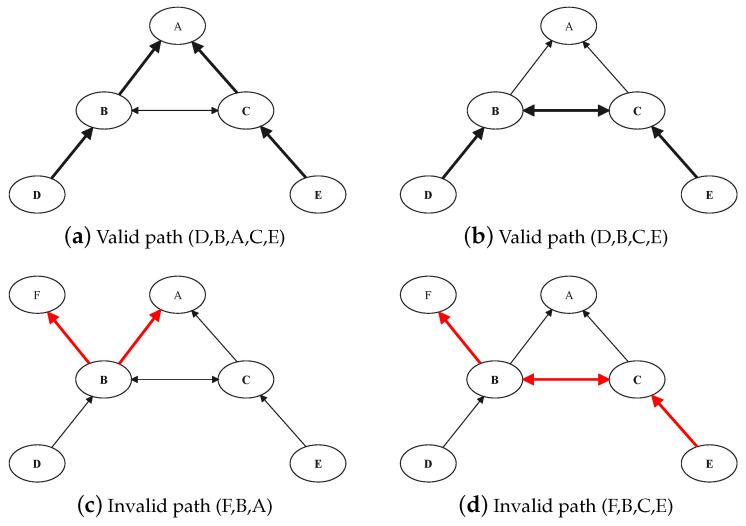
Valid path and invalid path.

Figure 2 shows several common path modes, with the bold line being the current path. The path in Figure 2a is (D,B,A,C,E). Among them, B, A, and C are relay AS nodes, and their customers are D, B and C, and E, respectively. Therefore, (D,B,A,C,E) are valid paths. Similarly, (D,B,C,E) in Figure 2b are valid paths. In Figure 2c, the relay node in the path (F,B,A) is B, while node B in the path has no customers connected to it, so (F,B,A) is an invalid path. Similarly, (F,B,C,E) in Figure 2d are also invalid paths. The general pattern of valid paths can be summarized from the examples: first, there are zero or more C2P, followed by zero or one P2P, and finally zero or more P2C.

### 3.2. Optimal Valid Path Discovery Algorithm Considering Business Relationships

When calculating the routing path, the general shortest path algorithm is not suitable for inter-domain routing networks.Therefore, calculating the routing path considering the business relationships needs a new algorithm to solve it.

Based on the two-way Dijkstra algorithm, this study proposed an optimal valid path discovery algorithm (DBR) that considers business relationships. The main process of the DBR is shown in Algorithm 1. The idea is that on the basis of two-way Dijkstra algorithm, the judgment of whether the path is connected is added to it, so that the overall complexity of the algorithm will not increase. DBR can finally determine whether there is a valid path between two nodes, and if so, output the optimal valid path.
**Algorithm 1** DBR**Input:** Predecessor predlist, successors succlist, complex network *G*, source, target, forward business relationships fcrlist, reverse business relationships rcrlist
**Output:** The optimalpath between the source node and the target node.
 1:forward_fringe.add(source) 2:reverse_fringe.add(target) 3:**while**forward_fringeandreverse_fringe**do** 4:   **if** len(forward_fringe) <= len(reverse_fringe) **then** 5:     this_level←forward_fringe, forward_fringe=[] 6:     **for** *v*∈this_level **do** 7:        **for** *w* ∈ Neighbour[v] **do** 8:          **if** *w*∉ pred **then** 9:             *k*← read_businessrelationship(G,v,w)10:            **if** (fcrlist[v]=1) or (fcrlist[v]=−1 and k=−1) or (fcrlist[v]=0 and k=−1) **then**11:               forward_fringe.add(w)12:               pred[w] ← *v*13:               d[w] ← *k*14:             **else**15:               w←v16:             **end if**17:          **end if**18:          **if** w∈succ **then**19:             **if** (fcrlist[w]=1) or (fcrlist[w]=−1 and p[w]=1) or (fcrlist[w]=0 and p[w]=1) **then**20:               optimalpath←pred+succ21:             **end if**22:          **end if**23:        **end for**24:     **end for**25:   **else**26:     this_level←reverse_fringe, reverse_fringe=[]27:     **for** v∈this_level **do**28:        **for** w∈Neighbour[v] **do**29:          **if** w∉succ **then**30:             j←read_businessrelationship(G,v,w)31:             **if** (rcrlist[v]=1) or (rcrlist[v]=−1 and j=−1) or (rcrlist[v]=0 and j=−1) **then**32:               succ[w]←v33:               reverse_fringe.add(w)34:               p[w]←j35:             **else**36:               w←v37:             **end if**38:          **end if**39:          **if** w∈succ **then**40:             **if** (rcrlist[w]=1) or (rcrlist[w]=−1 and fcrlist[w]=1) or (rcrlist[w]=0 and fcrlist[w]=1) **then**41:               optimalpath←pred+succ42:             **end if**43:          **end if**44:        **end for**45:     **end for**46:   **end if**47:**end while**


**Figure 3:**
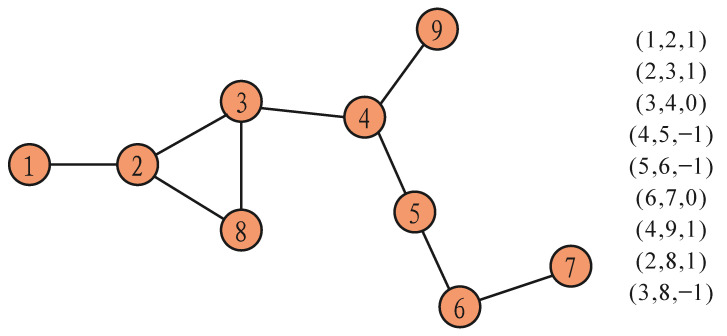
Simple network with business relationships.

In order to illustrate the effectiveness of the DBR algorithm, it was compared with the commonly used shortest path discovery algorithm [29] (Dijkstra) in a simple network. Figure 3 shows a simple network with business relationships. On the right is the business relationships between nodes, which is illustrated by (A,B,X). A and B are nodes, and X is the business relationships between A and B. If X = 1, then A and B are in a C2P relationship; if X = 0, then A and B are in a P2P relationship; and if X = −1, then A and B are in a P2C relationship. A total of 36 routing paths were found through the Dijkstra algorithm, and a total of 25 routing paths were found through the DBR algorithm. The extra 11 paths were confirmed to be invalid after a check. All the 11 extra paths were invalid. For example, there was no routing path for the node pair (1,7) and (1,9), and the path calculated by the Dijkstra algorithm was an invalid path. Therefore, it can be explained that the DBR algorithm can correctly identify the routing path in inter-domain routing systems.

## 4. IKN-CF

**Figure 4:**
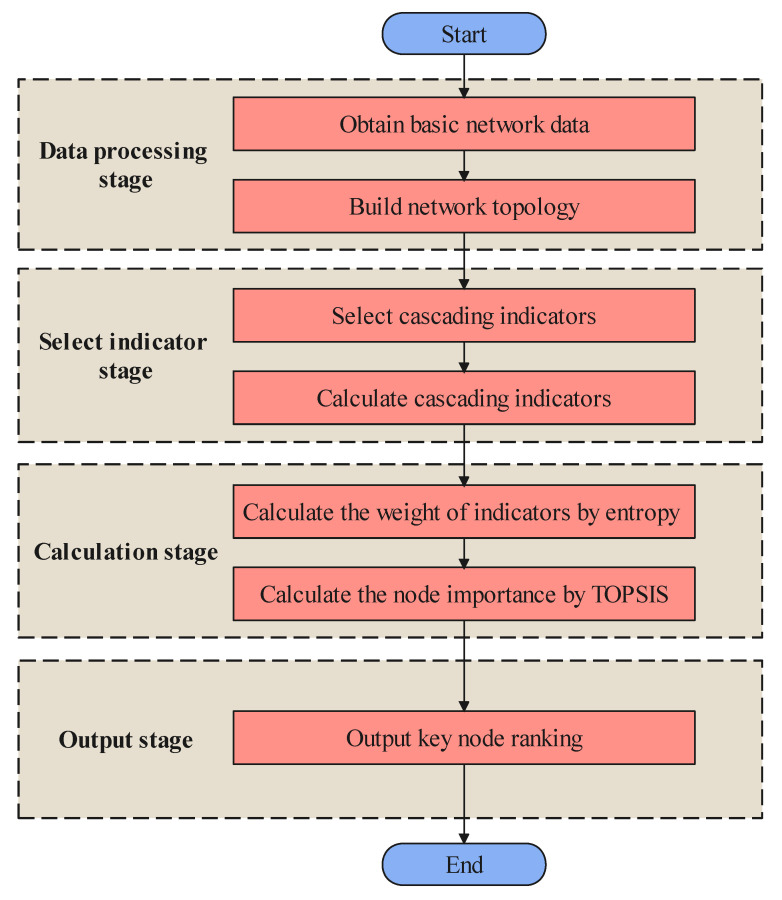
The process of IKN-CF.

As shown in Figure 4, IKN-CF mainly consists of four steps. The first step is to obtain basic network data, including business relationships and connection relationships. Then, the network topology is constructed to facilitate the calculation of the indicator. The second step is to select the cascading indicator that reflects the importance of the node, which is used to approximate the impact on the network after the node fails. The third step is to construct a key node identification model, including calculating the weight of the index and applying the TOPSIS method to obtain the importance value of the node. Finally, the ranking of importance of nodes is obtained. The important steps are described in detail below.

### 4.1. Selection of Cascading Indicators

#### 4.1.1. Analysis of Cascading Indicators

In inter-domain routing systems, it is generally considered that the cascading impact of node failure on the network determines the importance of node in the network. The importance of nodes increases as this influence increases. Therefore, the primary problem of identifying key nodes is how to approximate the influence of the cascading-failures process on nodes and links in the network after node failure. It can be seen from the analysis of cascading failures in our previous work [30], due to the cascading failures, that the failure of nodes in the inter-domain routing systems will have two effects on the network. On the one hand, the load of other nodes increases due to the propagation of UPDATE messages. If the load exceeds the capacity, the node fails. On the other hand, the load traffic of the failed node selects a new path to transmit information, and the load traffic of the selected link will increase. If it is greater than the capacity of the link, the link will also fail. The UPDATE messages propagation process corresponds to the change of control plane traffic, and the load redistribution process corresponds to the change of data plane traffic.

**Figure 5:**
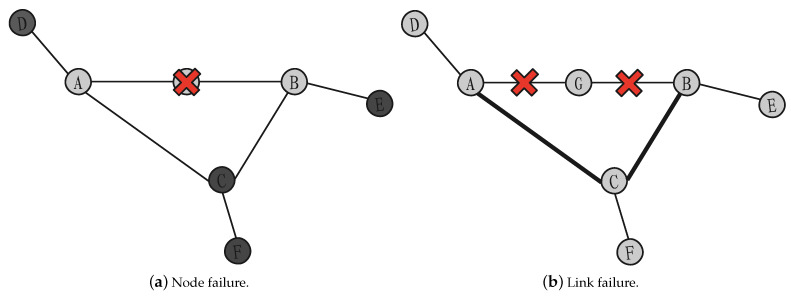
The impact of node and link failure.

Figure 5 is used to illustrate the effect of node and link failure. The heavier colors of nodes or links in the figure are caused by increased loads. In Figure 5a, after node G fails, neighbor nodes A and B will generate UPDATE messages, and reachable nodes C, D, E, and F of node G will receive UPDATE messages to increase the load. Afterwards, it is judged whether the number of UPDATE messages arriving at the same time is overloaded, and the overload becomes invalid. In Figure 5b, after node G fails, link AG and BG through node G are disconnected, and the traffic load that originally passed through node G will be rerouted to link AC and BC. In this case, the load of link AC and BC increases. The overload state also becomes invalid. Therefore, how to select reference indicators to approximate the impact of these two aspects on the network is the primary problem to be solved in evaluating the importance of nodes.

In addition to the changes in the node or link status during the cascading-failures process, the greatest change is the change in the traffic in the network. It is precisely because of changes in flow that failure will be caused. It can be seen from the above examples that the more reachable nodes of a failed node, the more UPDATE messages generated by neighbor nodes after the failure. When the number of reachable nodes of a node’s neighbor reaches a certain scale, it can cause traffic congestion on the control plane, which is more likely to cause cascading failures. Therefore, the number of reachable nodes of the neighboring node was selected to indicate the impact of the node failure on the control plane. For the load redistribution process, data plane traffic overload is the direct cause of load redistribution. The heavier the link load is, the more data traffic the node forwards. When the node fails, a large number of loads are rerouted to other links, which may lead to load redistribution and cascading failures. Therefore, the number of link loads was selected to indicate the impact on the data plane after the node fails.

The number of reachable nodes (NR) and the number of link loads (NL) are called cascading indicators, which can approximate the impact of node failure on the network. These two indicators are both positive indicators, which are positively related to the importance of nodes.

#### 4.1.2. Calculation of Cascading Indicators

From the above analysis, NR is used to represent the impact of node failure on the network data plane. The NR can be defined as:

(1) $$NR_{i}=\sum_{j\in \Gamma_{i}}R_{j},$$

where $\Gamma_{i}$ is the neighbor node set of $v_{i}$, and $R_{j}$ represents the reachable node of $v_{j}$, which can be obtained by the DBR algorithm presented in Section 3.2. The larger the NR of a node is, the more important the node is.

NL was used to represent the influence of node failure on the network control plane. NL can be defined as:

(2) $$NL_{i}=\sum_{j\in \Gamma_{i}}L_{ij},$$

where $L_{ij}$ is mainly related to the number of valid paths $N_{OVPS_{mn}\left(i,j\right)}$ calculated by the DBR algorithm and the unit traffic α on the link. $L_{ij}$ can be denoted as:

(3) $$L_{ij}=\sum_{\forall m,n\in V}\alpha N_{OVPS_{mn}\left(i,j\right)},$$

### 4.2. The Identifying Key Nodes Model

The weights for NR and NL cannot be obtained empirically. To be more objective and accurate, this study used entropy to calculate the weight of the indicator. The indicator that can reflect the importance of nodes is regarded as the scheme’s attributes, and the importance of each scheme is quantified by calculating the proximity to the best scheme of each attribute by TOPSIS. Finally, we can obtain the ranking of node importance.

$V=\{v_{1},v_{2},v_{3},\cdot \cdot \cdot ,v_{n}\}$ is the set of nodes, and $C=\{c_{1},c_{2},c_{3},\cdot \cdot \cdot ,c_{m}\}$ is the indicator set of nodes. The *j*-th indicator of the $v_{i}$ is denoted as $c_{i,j}\left(i=1,2,3,\cdot \cdot \cdot ,n;j=1,2,3,\cdot \cdot \cdot ,m\right)$. Then, the indicator matrix of the node can be expressed as $E={\left(c_{i,j}\right)}_{n\times m}$.

#### 4.2.1. Using Entropy to Calculate the Weight of Indicators

Generally, the information entropy of a certain indicator is smaller, and the variation of the indicator is greater. Therefore, the more information it can provide the greater its role in the TOPSIS and the greater its weight. In order to eliminate the impact of different orders of magnitude and units, we first needed to standardize the indicator matrix E, and then a normalized $U={\left(u_{i,j}\right)}_{n\times m}$ was obtained. The process can be denoted as:

(4) $$u_{i,j}=\frac{c_{i,j}-\min_{1\leq i\leq n}\left(c_{i,j}\right)}{\max_{1\leq i\leq n}\left(c_{i,j}\right)-\min_{1\leq i\leq n}\left(c_{i,j}\right)},$$

where i=1,2,3,···,n;j=1,2,3,···,m, $u_{i,j}$ is the normalized value of the *j*-th indicator of $v_{i}$. $\max_{1\leq i\leq n}\left(c_{i,j}\right)$ is the maximum value of the *j*-th indicator. $\min_{1\leq i\leq n}\left(c_{i,j}\right)$ is the minimum value of the *j*-th indicator.

Then, we calculated the information entropy $H_{j}$ of the *j*-th indicator in the *U*, and $H_{j}$ can be denoted as:

(5) $$H_{j}=-\frac{\sum_{i=1}^{n}a_{i,j}\ln a_{i,j}}{\ln n},$$

where j=1,2,3,···,m. When $a_{i,j}=0$, $a_{i,j}\ln a_{i,j}=0$, $a_{i,j}$ can be denoted as:

(6) $$a_{i,j}=\frac{u_{i,j}}{\sum_{i=1}^{n}u_{i,j}},$$

The weight of the *j*-th indicator can be denoted as:

(7) $$W_{j}=\frac{1-H_{j}}{m-\sum_{i=1}^{m}H_{j}},$$

where j=1,2,3,···,m.

Finally, the weight vector indicator was determined as $W={\left(W_{1},W_{2},W_{3},\cdot \cdot \cdot ,W_{m}\right)}^{T}$, and $\sum_{j=1}^{m}W_{j}=1$.

#### 4.2.2. Using TOPSIS to Calculate the Importance of Nodes

Firstly, the weighted normalized indicator matrix $E={\left(p_{i,j}\right)}_{n\times m}$ was constructed from *W* and *U*. The calculation process can be denoted as:

(8) $$E={\left(p_{i,j}\right)}_{n\times m}={\left(W_{j}\cdot u_{i,j}\right)}_{n\times m}i\in n,j\in m,$$

where $p_{i,j}$ is the element of the weighted normalized evaluation matrix *E*, and $W_{j}$ is the weight corresponding to the attribute. $u_{i,j}$ is the element of the normalized evaluation matrix *U*.

Secondly, to find out the positive ideal solution $p^{+}=\left\{p_{1}^{+},p_{2}^{+},p_{3}^{+},\cdot \cdot \cdot ,p_{m}^{+}\right\}$ and the negative ideal solution $p^{-}=\left\{p_{1}^{-},p_{2}^{-},p_{3}^{-},\cdot \cdot \cdot ,p_{m}^{-}\right\}$ for each indicator, the calculation process can be denoted as:

(9) $$\left\{\begin{array}{c} p_{j}^{+}=\left\{p_{1}^{+},p_{2}^{+},p_{3}^{+},\cdot \cdot \cdot ,p_{m}^{+}\right\}=\max_{i}\left\{p_{i,j}|1\leq i\leq n\right\}\\ p_{j}^{-}=\left\{p_{1}^{-},p_{2}^{-},p_{3}^{-},\cdot \cdot \cdot ,p_{m}^{-}\right\}=\min_{i}\left\{p_{i,j}|1\leq i\leq n\right\}, \end{array}\right.$$

where j=1,2,3,···,m, and $p^{+}$ is the maximum value of the *j*-th indicator. $p^{-}$ is the minimum value of the *j*-th indicator.

Then, the weighted distance between each attribute and its positive ideal point $D_{i}^{+}$ and negative ideal point $D_{i}^{-}$ in the weighted normalized evaluation matrix is solved. The calculation process can be denoted as:

(10) $$\left\{\begin{array}{c} D_{i}^{+}=\sqrt{\sum_{j=1}^{m}W_{j}*{\left(p_{j}^{+}-p_{i,j}\right)}^{2}}\\ D_{i}^{-}=\sqrt{\sum_{j=1}^{m}W_{j}*{\left(p_{j}^{-}-p_{i,j}\right)}^{2}} \end{array}\right.,i=1,2,3,\cdot \cdot \cdot ,n.$$

Finally, the importance value of each node is calculated, that is, the degree of closeness to the ideal point $Q=\{q_{1},q_{2},q_{3},\cdot \cdot \cdot ,q_{n}\}$. The calculation process can be denoted as:

(11) $$z_{i}=\frac{D_{i}^{-}}{D_{i}^{+}+D_{i}^{-}},i=1,2,3,\cdot \cdot \cdot ,n,0\leq z_{i}\leq 1,$$

where $z_{i}$ is the proximity of $v_{i}$ to the ideal point. The $z_{i}$ is larger, the closer the evaluation attribute of $v_{i}$ is to the positive ideal point, and its importance is higher. On the other hand, the $z_{i}$ is smaller, the farther the evaluation attribute of $v_{i}$ is from the positive ideal point, and its importance is lower.

## 5. Results and Analysis

### 5.1. Data and Parameters in the Experiment

The cascading-failures model for inter-domain routing systems (CFM-IRS) proposed by Zhang et al. [31] is the most reasonable and realistic model for modeling and analyzing cascading failures of inter-domain routing systems. In order to ensure the fairness and accuracy of the comparative experiment, the experiment in this study was carried out in CFM-IRS. The topological structures of India and UK were selected from the as-relationships data set of the CAIDA [32] project in August 2021. We analyzed the connection and business relationships between AS to establish a basic network environment as the basis for running the CFM-IRS. The information for India and the UK is shown in Table 2. It includes parameters related to the CFM-IRS running.

### 5.2. Metric of Evaluation

In this study, the failure rate was used to evaluate the damage degree of the network. The greater the failure rate, the greater the damage of the network. The Kendall tau correlation coefficient [17] was used to calculate the correlation between the two ranking lists. When the Kendall tau correlation coefficient approaches 1, it indicates that the two ranking lists are very similar.

#### 5.2.1. Failure Rate

A node’s importance is generally considered equivalent to the amount of damage to the network caused by removing the node. The failure rate (FR) was used to indicate the degree of damage to the network caused by the removal of nodes, which can be denoted as:

(12) $$f=\frac{N_{Fv}+N_{Fe}}{W},$$

where $N_{Fv}$ represents the number of failed nodes. $N_{Fe}$ represents the number of failed links. $N_{Fv}+N_{Fe}$ represents the number of all failures in the network. *W* represents the total number of initial nodes and links.

The greater the FR of $v_{i}$, the higher the importance of $v_{i}$. It is difficult to obtain the FR of all nodes directly in a large-scale network, due to the complexity of CFM-OVP running. However, a small number of nodes were given, and a local optimal ranking (LOR) was obtained according to the failure rate in descending order. LOR can be used as a standard for evaluating the method of key nodes.

#### 5.2.2. Kendall Tau Correlation Coefficient

In order to evaluate the accuracy of IKN-CF, the Kendall tau correlation coefficient (KT) was used to measure the degree of correlation between the methods. It can be denoted as:

(13) $$\tau =\frac{N_{c}-N_{d}}{\frac{1}{2}N\left(N-1\right)},$$

where $N_{c}$ is the number of concordant pairs, $N_{d}$ is the number of discordant pairs, and *N* is the number of nodes in the network. Let $\left(x_{1},y_{1}\right),\left(x_{2},y_{2}\right),\cdot \cdot \cdot ,\left(x_{n},y_{n}\right)$ be a joint node pair of two ranking *X* and *Y*. If $x_{i}>x_{j}$ and $y_{i}>y_{j}$ or $x_{i}<x_{j}$ and $y_{i}<y_{j}$, the $\left(x_{i},y_{i}\right)$ and $\left(x_{j},y_{j}\right)$ are considered to be the concordant pairs. If $x_{i}>x_{j}$ and $y_{i}<y_{j}$ or $x_{i}<x_{j}$ and $y_{i}>y_{j}$, the $\left(x_{i},y_{i}\right)$ and $\left(x_{j},y_{j}\right)$ are considered to be the discordant pairs. If $x_{i}=x_{j}$ or $y_{i}=y_{j}$, the $\left(x_{i},y_{i}\right)$ and $\left(x_{j},y_{j}\right)$ are considered to be neither the concordant pairs nor discordant pairs.

### 5.3. Experimental Analysis

#### 5.3.1. Effectiveness of IKN-CF

**Figure 6:**
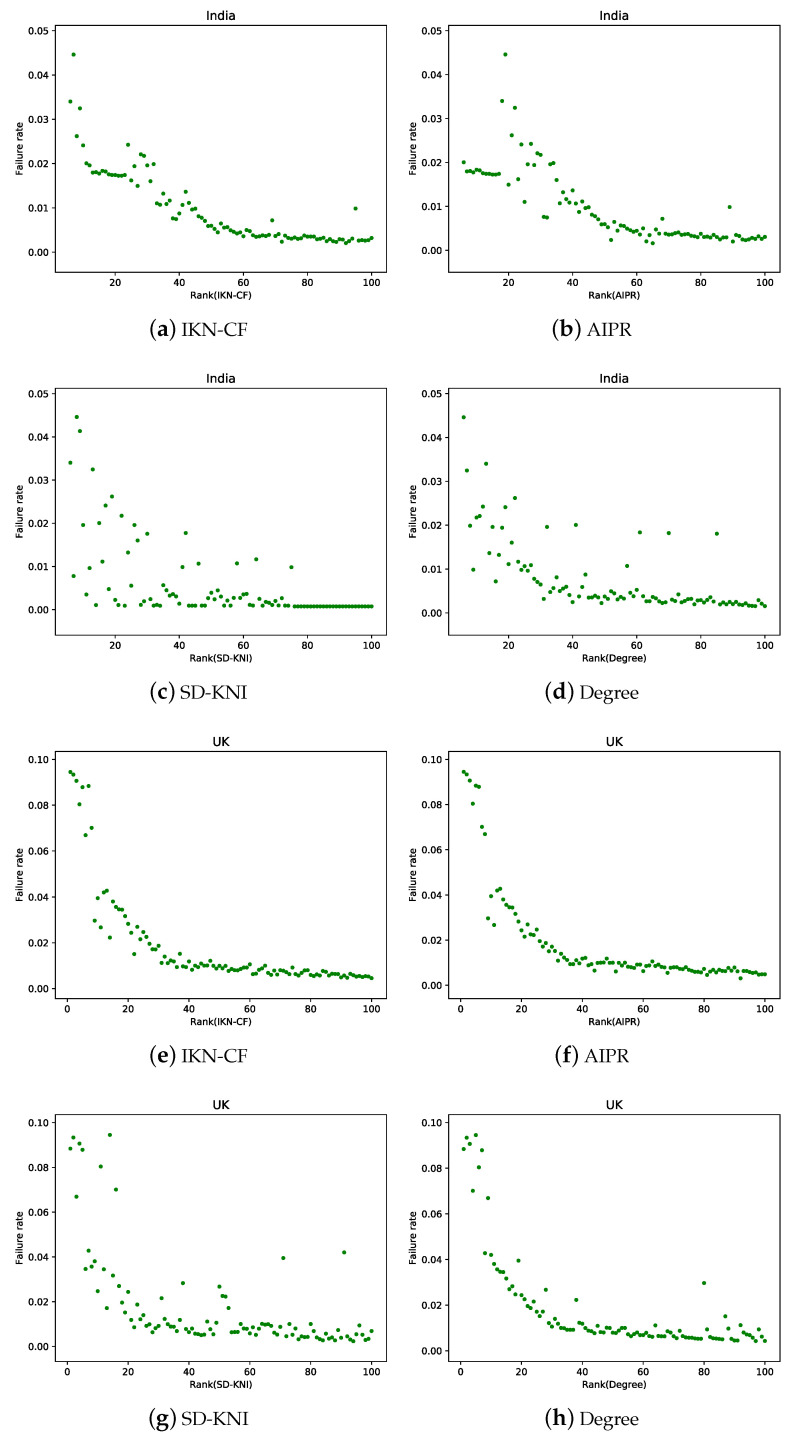
The relationship between the top 100 key nodes and the FR after nodes are removed. The green dots are nodes in the network, and the X-axis of the dots indicates the ranking number of the node under the corresponding method. The Y-axis of the dot indicates the failure rate of after it had been removed.

In order to verify the accuracy of the method, only one node was removed from the inter-domain routing network at one time, and the FR of the network after the removal of this node was calculated. Until all nodes had been individually removed once, we calculated the FR of each node after being removed separately. In this study, AIPR [26], spreading-dynamics-based key nodes identification (SD-KNI) [27], and degree centrality [10] were compared with IKN-CF. Figure 6 shows the relationship between the importance ranking of nodes and FR obtained by the four ranking methods in the two inter-domain routing networks. The lower the number of the X-axis, the more influential the node was. Since there is little difference in the importance of the nodes at the bottom of the ranking, only the results of the top 100 in the ranking are shown in Figure 6.

As shown in Figure 6, in the Indian network, the importance ranking result of IKN-CF proposed in this study had the best effect compared with other methods, and the FR basically decreased with the increase in the importance ranking number. The AIPR resulted in a ranking with the first 20 nodes, which is obviously not reasonable. SD-KNI had the worst ranking result for the importance of nodes, and the importance of nodes after ranking number 76 was consistent, indicating that the was is not accurate and that the method is relatively rough. As shown in Figure 6d, the overall trend of degree ranking results was better than AIPR and SD-KNI, but the ranking results were somewhat scattered. In the UK network, the results of the AIPR and IKN-CF were reasonable. As shown in Figure 6g, the SD-KNI ranking result was the most chaotic. According to the ranking obtained by the degree, there were more nodes with high importance in the ranking numbers between 80 and 100, as shown in Figure 6h. We found failure rate faults with the decrease in the importance of nodes, indicating that the nodes with extensive damage to the network were more concentrated.

#### 5.3.2. Accuracy of IKN-CF

**Figure 7:**
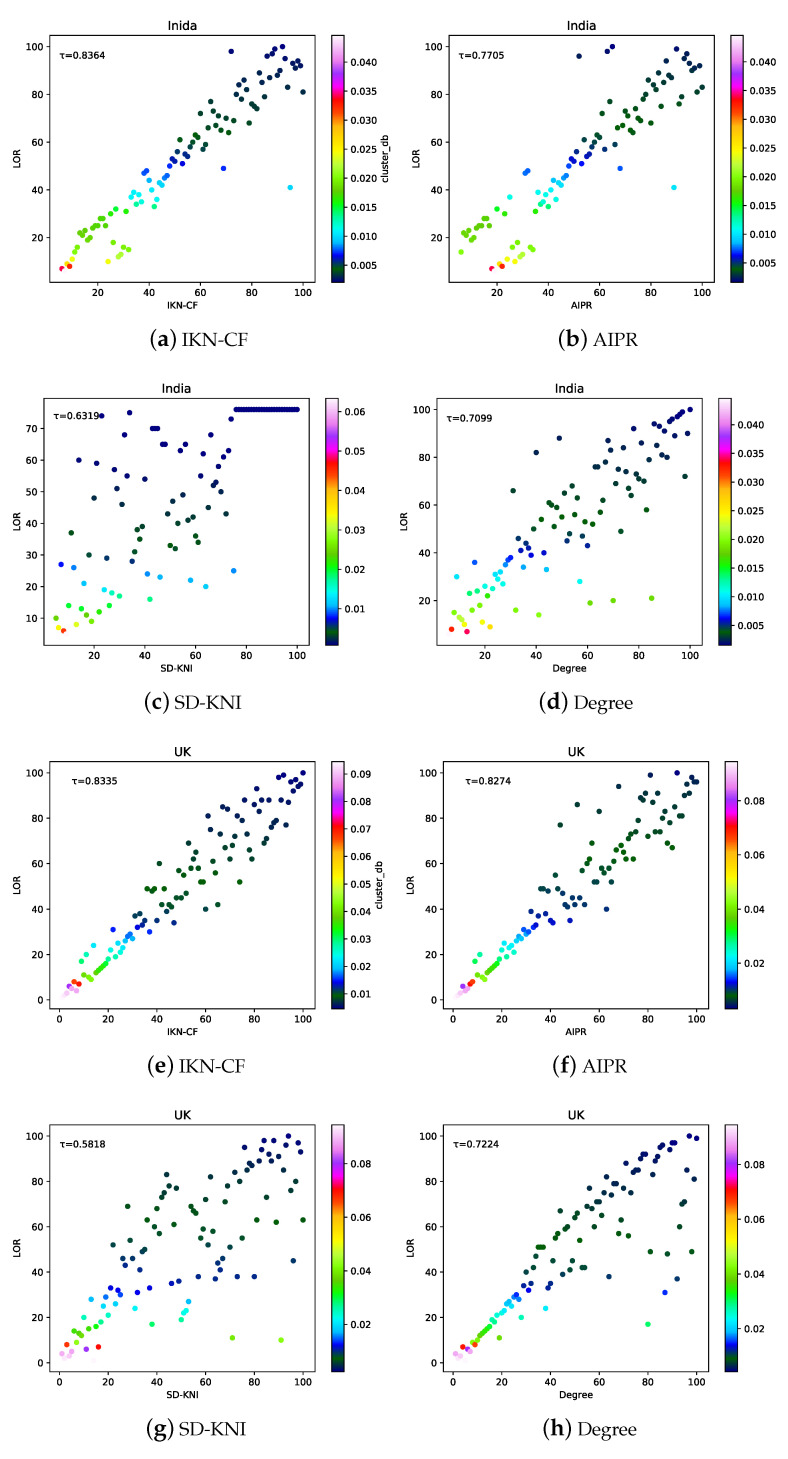
The KT between LOR and results of the top 100 in the ranking obtained by the IKN-CF, AIPR, SD-KNI, and degree on two real networks (India and UK). The X-axis of the dots indicates the ranking number of the node under the corresponding method. The Y-axis of the dot indicates the ranking number of the node in LOR. The color of the dot indicates the failure rate after removing the node.

To further compare the accuracy of the methods, the KT between the results of the top 100 in the ranking obtained by the four methods and LOR was calculated in the two networks, as shown in Figure 7. In the Indian network, the $\tau_{KT}=0.8364$ between IKN-CF and LOR, which was the highest among the four methods. It shows that the ranking result of IKN-CF is the most similar to the LOR and is the most accurate among the four methods. The KT between SD-KNI and LOR was the lowest, being only 0.6319. In addition, the closer the position of the colored dots to the diagonal line, the better the ranking result. As can be seen from the distribution of the colored dots in Figure 7a–d, IKN-CF was superior to AIPR, SD-KNI, and degree. SD-KNI and degree often had a ranking result that did not match the importance of the nodes, which is obviously inappropriate.

In the UK network, the $\tau_{KT}=0.8335$ between IKN-CF and LOR, which was still the best among the four methods. The $\tau_{KT}=0.8274$ between AIPR and LOR, and it as similar to IKN-CF. This shows that the two methods were equally accurate in the UK. As shown in Figure 7h, by observing the position and color of the dots, the ranking result of degree was the most accurate at the nodes with a ranking number between 10 and 20.

Overall, IKN-CF had a similar KT in the two networks, and it was the highest among the four methods, indicating that IKN-CF is stable, widely applicable, and accurate. However, SD-KIN not only has a poor ranking effect but also fluctuates wildly in different networks. The ranking result of AIPR was second only to IKN-CF and superior to SD-KNI and degree. For the degree, the importance ranking ability of nodes with the ranking number after 30 needs to be improved.

## 6. Conclusions

For the shortcomings of traditional identification methods for important nodes in inter-domain routing systems, this study proposed a method for identifying key nodes in inter-domain routing systems based on cascading failures. In addition, based on the existing business relationships of inter-domain routing systems, this study proposed an optimal valid path discovery algorithm, which provides convenience for the calculation of cascading indicators. In the two inter-domain routing systems (India and the UK), we compared the top 100 in the ranking obtained by the IKN-CF, AIPR, SD-KNI, and degree. The experimental results show that the IKN-CF performed best, and the failure rate basically decreased with the increase in the importance-ranking number. Then, the KT was introduced. In the Indian network, the $\tau_{KT}=0.8364$ between IKN-CF and LOR. In the UK network, the $\tau_{KT}=0.8335$ between IKN-CF and LOR. Compared with the other three methods, IKN-CF had the highest KT with LOR, with an average increase of at least 12.8%. In addition, the KT of IKN-CF in the two networks was almost the same, which indicates that the IKN-CF is stable and widely applicable.

In future work, under the premise of considering the cost, we plan to analyze the damage strategy that the attacker may apply to provide a basis for defending the inter-domain routing systems. The IKN-CF can provide significant support for this.

## Figures and Tables

**Table 1:** Commonly used notations.

Notations	Descriptions
G=(V,E)	Topology of inter-domain routing network.
*V*	The set of nodes.
*E*	The set of links.
$v_{i}$	A node $v_{i}\in V$.
$e_{ij}$	A link $e_{ij}\in E$.
$L_{i}/L_{ij}$	The load of $v_{i}$ or $e_{ij}$.
$R_{i}$	The capacity of $v_{i}$.
$C_{ij}$	The capacity of $e_{ij}$.

**Table 2:** Parameter setup.

Parameter	India	UK	Descriptions
Num_V	2406	1191	The number of nodes.
Num_E	4052	2946	The number of links.
α	1	1	Basic unit flow.
β	0.3	0.3	Tolerance parameter.
R	250	100	The capacity of $v_{i}$.
$\Delta T_{i}$	5	5	Recovery delay of $v_{i}$.
$\Delta T_{ij}$	5	5	Recovery delay of $e_{ij}$.

## Data Availability

The data used in this study are from https://publicdata.caida.org/datatasets/as-relationships/ (accessed on 25 September 2021).
